# Supplementary material for: Lung Organotypic Slices Enable Rapid Quantification of Acute Radiotherapy Induced Toxicity
Source: Cells. 2023 Oct 11;12(20):2435. doi: 10.3390/cells12202435 (PMC10605600; doi:10.3390/cells12202435)
Supplement: Supplementary file 1 [file cells-12-02435-s001.zip › cells-2637177-supplementary.pdf]

Supplementary Materials:

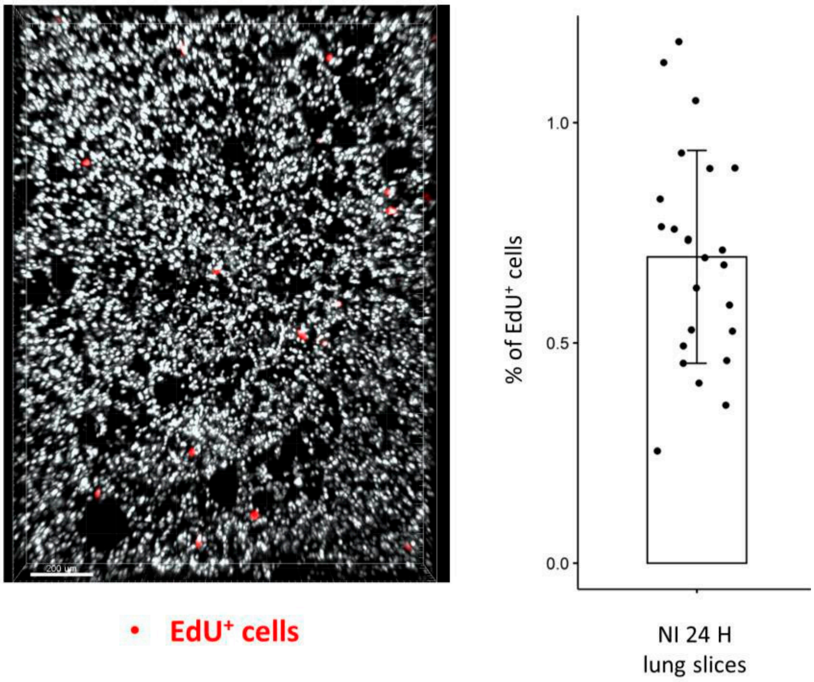

Supplementary Figure S1. 3D reconstruction and quantification of the proportion of EdU^{+} cells in one observation field compared to the total of cells in one organotypic lung slices.

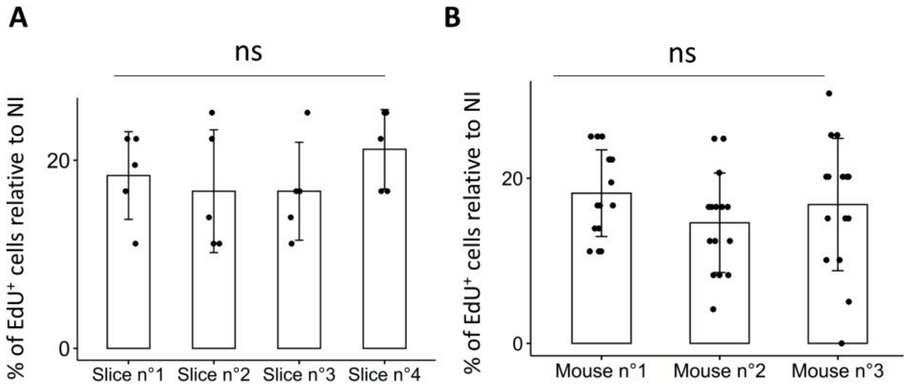

Supplementary Figure S2. Evaluation of the variability of the proportion of EdU+ among different slices and mice cells between slices (A) Absence of a significant difference between slices obtained from the same mouse. EdU+ cells were counted 24 hours after irradiation with a dose of 9 Gy (5 FOV per slice). (B) Quantification of the proportion of EdU^{+} cells from organotypic lung slices obtained from three mice 24-hours after exposure to 9 Gy. No significant (ns) difference was found between the mice analyzed.

FLASH beam parameters			
Beam energy [MeV]	7		
Total absorbed dose [Gy]	3	6	9
Number of pulses	1	2	3
Pulse frequency [Hz]	100		
Mean dose rate [Gy/s]	7.5E+05	600	450
Instantaneous dose rate [Gy/s]	7.5E+05	7.5E+05	7.5E+05
Dose per pulse [Gy]	3		
Pulse width [µs]	4		
Duration of exposure [ms]	0.004	10	20
Homogeneous field size$_{+/-\ 2\%}$ [cm]	11		

Supplementary Table S1. Beam parameters used for FLASH radiation of organotypic lung slices.
